# Proximal femoral anatomy and collared stems in hip arthroplasty: is a single collar size sufficient?

**DOI:** 10.1186/s40634-017-0107-3

**Published:** 2017-10-03

**Authors:** Nicolas Bonin, Jean-Emmanuel Gedouin, Vincent Pibarot, Jacques Bejui-Hughues, Hugo Bothorel, Mo Saffarini, Cécile Batailler

**Affiliations:** 1 Lyon Ortho Clinic, 29B Avenue des sources, Lyon, France; 2 Nouvelles Cliniques Nantaises - Le Confluent, 3 rue Tabarly, Nantes, France; 3 Service de chirurgie orthopédique et traumatologique, Hôpital Edouard Herriot, Lyon, France; 4 Istituto Chirurgico Ortopedico Traumatologico ICOT, Latina, Italy; 5 ReSurg SA, Chemin de la Vuarpillière 35, 1260 Nyon, Switzerland; 6 Hospices Civils de Lyon, Lyon, France

**Keywords:** Total hip arthroplasty, Collared stem, Prosthetic overhang, Iliopsoas impingement, Stem subsidence, Preoperative planning

## Abstract

**Background:**

Even if the benefits of collars are unclear, they remain widely used, in several femoral stem designs. This study aimed to determine whether collar size should be proportional to hip dimensions and morphology. The hypothesis was that the collar should be larger for greater stem sizes and for varus femoral necks.

**Methods:**

Computed Tomography scans of 204 healthy hips were digitally analysed and manually templated to determine principle dimensions, appropriate stem size and model, as well as cortical distance at the femoral calcar (ideal collar size).

**Results:**

Univariable analysis revealed that cortical distance was moderately correlated with mediolateral offset (*r* = 0.572; *p* < 0.0001) and stem model (*r* = 0.520; *p* < 0.0001). Cortical distance was weakly correlated with head diameter (*r* = 0.399; *p* < 0.0001), stem size (*r* = 0.200; *p* = 0.017), and patient gender (*r* = 0.361; *p* < 0.0001). Multivariable analysis confirmed that stem model (*p* < 0.0001) and head diameter (*p* = 0.0162) are directly correlated to cortical distance.

**Conclusion:**

We found that cortical distance along the femoral calcar is directly correlated with the model of the stem implanted (‘standard’ or ‘varus’) and with the head diameter. This cortical distance indicates optimal collar size, which would grant maximum calcar coverage without prosthetic overhang. Collar size should be proportional to the size of the operated hip, and should be larger for ‘varus’ stem models than for ‘standard’ stem models.

## Background

The advent of uncemented Total Hip Arthroplasty (THA) required alternative implant features to grant initial stability and stimulate long-term osteo-integration. The addition of collars to femoral stems was intended to enable load transfer to the resected femoral calcar, and thereby prevent implant subsidence within the cancellous bone of the metaphysis (Demey et al., 2011; Flecher et al., 2012).

Since their development, collars have been controversial, with unclear evidence of their benefits. Several authors investigated the benefits and drawbacks of collared stems and found little or no differences, in either short- or long-term outcomes, when compared to collarless stems (Al-Najjim et al., 2016; Caglar et al., 2008; Ebramzadeh et al., 2004; Lenart et al., 2012; Weber et al., 2014). Conversely, a number of clinical studies supported the use of collars and argued that they could improve stem survival and facilitate revision THA (Flecher et al., 2012; Kale et al., 2000; Van Kleunen et al., 2006). Furthermore, good collar-calcar coverage could prevent stem subsidence and rotation, which may occur during the first weeks following uncemented THA (Campbell et al., 2011; Parvizi et al., 2004; Simpson et al., 2010; Strom et al., 2007; Weber et al., 2014).

**Fig. 1:**
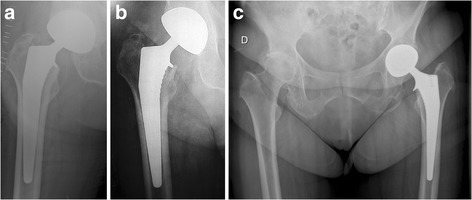
**a** Immediate post-operative and **b** 2 months post-operative X-rays of a right hip after total hip arthroplasty with insufficient collar size, showing slight subsidence with signs of calcar fracture. **c** Post-operative X-ray of a left hip illustrating an oversized collar, which may cause impingement on surrounding soft tissues

The efficacy of a collar depends on how well it covers the femoral calcar (Demey et al., 2011; Fischer et al., 1992; Jeon et al., 2011; Keaveny & Bartel, 1993; Mandell et al., 2004). While an undersized collar may be insufficient to prevent stem subsidence or rotation (Fig. 1a and b) (Meding et al., 1997), an oversized collar may lead to painful prosthetic impingement against the ilipsoas or other soft tissues (Fig. 1c) (Brew et al., 2011; Lindner et al., 2013). To the authors’ knowledge, however, there are no published studies that investigated optimal collar dimensions in relation to stem size or neck angle.

Even if its benefit remains unclear, the collar is widely used in current femoral stems. The purpose of the present study was to determine whether collar size should be proportional to the dimensions and morphology of each hip. The hypothesis was that the collar should be larger for greater stem sizes and for varus femoral necks. Such findings could help implant manufacturers adjust the dimensions of the collar as required for each implant size and standard/varus model.

## Methods

The authors studied 204 Computed Tomography (CT) hip scans taken for femoral angiography at a cardiology centre in 2014. The images were all acquired using the same scanner (Philips Brilliance 64, Amsterdam, The Netherlands) with 2 mm slice thickness, 1 mm inter-slice distance and reconstructions in the coronal plane. All scans were viewed in standard resolution and included the pelvis and the proximal half of the femur. Patients with arthritic or orthopaedic pathologies in either of their hips were excluded (*n* = 1).

The scans were then analysed using image-processing software to convert the DICOM images to three-dimensional (3D) reconstructions (Invesalius, Campinas, Brazil). The ideal resection planes for stem positioning were determined using the engineering programme Creo (Parametric Technology Corporation, Needham, MA, USA) which calculated the 3D coordinates of (i) the femoral head centre and diameter by fitting a ‘sphere of best fit’, (ii) the proximal diaphyseal axis, and (iii) the femoral neck axis.

**Fig. 2:**
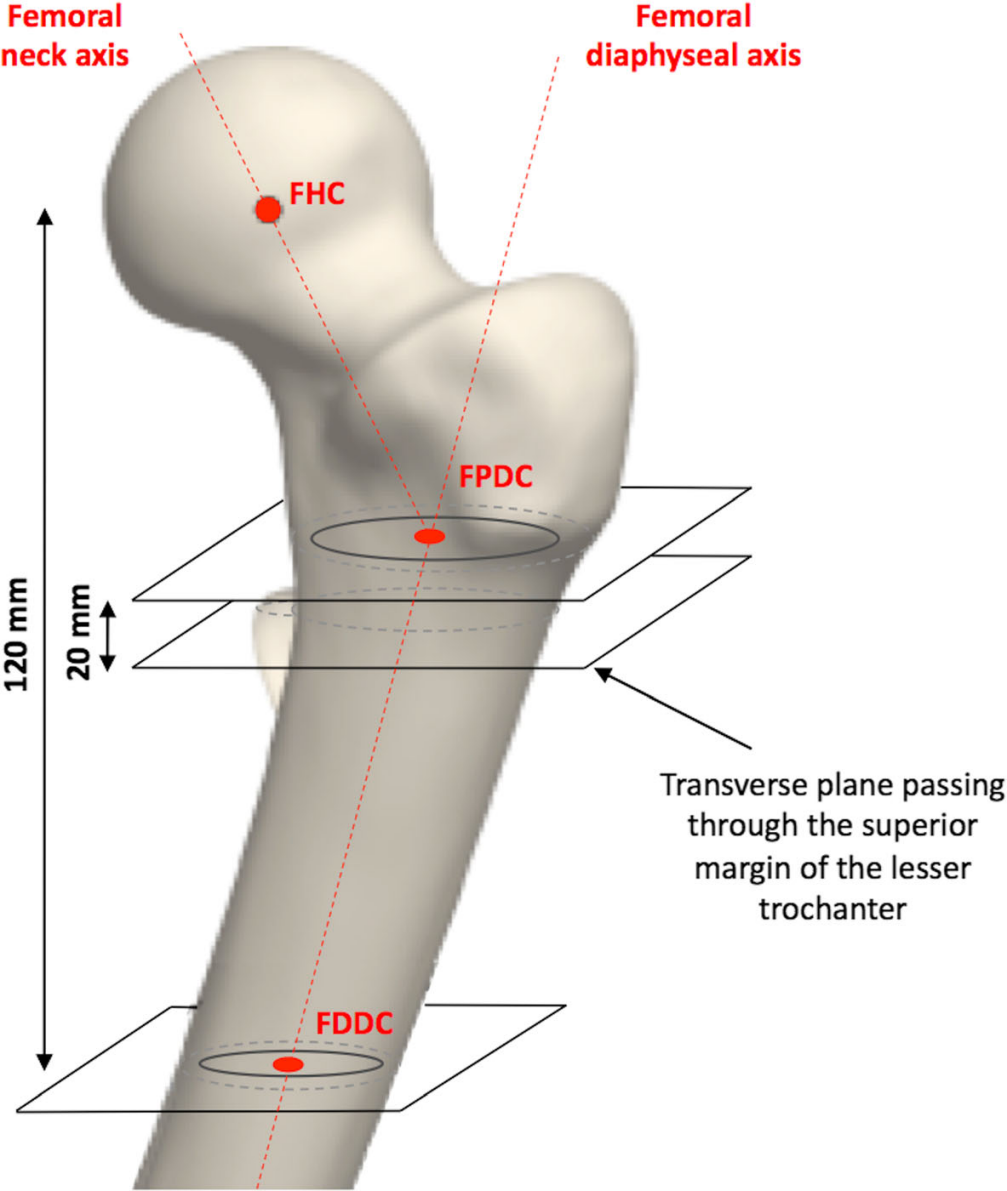
Anatomic landmarks, axes and dimensions determined for each hip. The Femoral Head Center (FHC) is determined by fitting a ‘sphere of best fit’ to the femoral head. The Femoral Distal Diaphysis Center (FDDC) is determined by fitting an ellipse to the intramedullary cortex 120 mm below the FHC. The Femoral Proximal Diasphysis Center (FPDC) is determined by fitting an ellipse to the intramedullary cortex 20 mm above the superior margin of the lesser trochanter

**Fig. 3:**
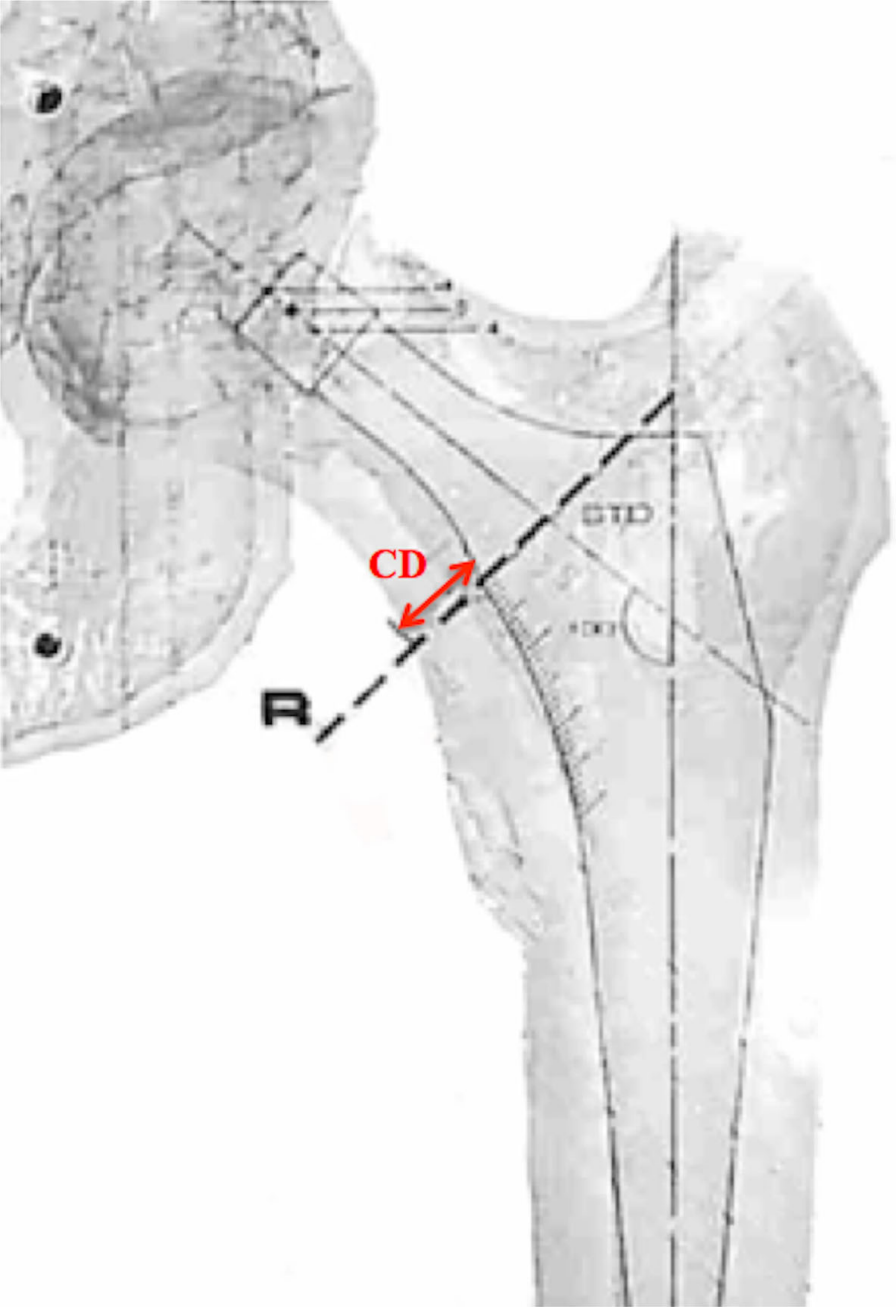
Templating femoral stems on printed CT-scans to select the size and model of the stem matched and to measure the cortical distance (CD) at the level of the collar resection (femoral calcar)

The true frontal views of 204 femurs were then printed, with magnification of 115%, accounting for femoral neck anteversion. This view corresponded to the plane passing through 3 points: (a) the femoral head centre, (b) the femoral distal diaphysis centre at 120 mm below the femoral head centre, and (c) the femoral proximal diaphysis centre 20 mm proximal to the superior margin of the lesser trochanter (Fig. 2). The engineering software then used the true frontal view to automatically calculate the femoral head diameter, the femoral neck angle (FNA), between the neck axis and the diaphyseal axis, and the medio-lateral femoral offset between the femoral head centre and the diaphyseal axis. A total of four surgeons (three senior and one junior) fitted these true frontal views with templates of a collared femoral stem (Symbol®, Dedienne Santé, Mauguio, France) also printed with magnification of 115%. The implant is delivered in ten sizes (1–10) with each available in ‘standard’ and ‘varus’ models (femoral neck angles 130° and 120°, respectively). For each hip, the surgeons noted the size and model of the stem that best fitted the femur and maintained the native head centre, without considering the acetabulum. The Cortical Distance (C-D) was then measured using a ruler with 0.5 mm graduation, at the level of the collar, between the medial margin of the stem and the outer cortex of the femoral calcar (Fig. 3). The template fitting was performed twice for each hip, by two different surgeons, to enable calculation of inter-observer repeatability of all variables.

### Statistical analysis

Statistical analyses were performed using R version 3.3.2 (R Foundation for Statistical Computing, Vienna, Austria). Descriptive statistics were used to summarize the data. The inter-observer agreement was good for the choice of a ‘standard’ or ‘varus’ stems (Kappa coefficient, 0.685), satisfactory for the size of the chosen stem (Kendall’s tau, 0.797), and satisfactory for measurements of cortical distance (Kendall’s tau, 0.642). For non-Gaussian quantitative data, between group differences were evaluated using Wilcoxon rank sum tests (Mann Whitney U test). For continuous variables, correlations were analysed using Pearons coefficients while for categoric variables, correlations were studied using Spearman coefficients. Step-wise descending multivariable linear and logistic regression were performed. Models assumptions were checked before the analyses were performed. *P*-values <0.05 were considered statistically significant.

## Results

The scans studied comprised 150 male hips (73.5%) and 54 female hips (26.5%) aged 68.5 ± 12.2 years (range, 35–93 years). The mean cortical distance measured was 10.2 ± 2.6 mm (range, 3.0–24.0 mm). The mean FNA was 125.3° ± 5.7° (range, 110.5°-140.8°), and ‘varus’ stem models were templated for 83 of the hips (40.7%). The selected stem size was small (1–3) for 44 hips (21.6%), medium (4–6) for 104 hips (51%) and large (7–10) for 56 hips (27.5%).

There were several significant differences observed between hips of different genders (Table 1). First, men were significantly younger than women (*p* < 0.0001), and had considerably larger absolute dimensions, including cortical distance, femoral head diameter, and medio-lateral offset (*p* < 0.0001). There were no significant differences, however, between men and women in terms of FNA, with equal proportions of ‘varus’ stems templated for both genders.

**Table 1:** Demographics and dimensions by gender

		Female hips (*n* = 54)					Males hips (*n* = 150)					*p*-value
		Mean	±SD	Median	Range		Mean	±SD	Median	Range		
Age	(yrs)	74.8	±12.6	77.0	(41.0	- 93.0)	66.2	±11.2	66.0	(35.0	- 89.0)	<0.001
Femoral Head Diameter	(mm)	43.8	±2.4	44.5	(38.0	- 48.0)	49.1	±2.6	49.0	(43.0	- 56.0)	<0.001
Cortical distance	(mm)	8.6	±2.0	9.0	(3.0	- 13.0)	10.7	±2.6	10.0	(5.0	- 24.0)	<0.001
Medio-lateral Offset	(mm)	40.5	±4.3	39.8	(31.3	- 51.2)	45.3	±5.1	45.1	(32.7	- 65.0)	<0.001
Stem size	(1–10)	3.7	±1.7	3.0	(1.0	- 8.0)	5.7	±1.7	6.0	(2.0	- 9.0)	<0.001
Femoral Neck Angle	(deg)	126.2	±5.8	125.9	(112.3	- 136.4)	124.9	±5.6	124.8	(110.5	- 140.8)	0.165
Varus Stem Model		40.7%					40.7%					

Univariable analysis revealed that the cortical distance was moderately correlated with medio-lateral offset (*r* = 0.572; *p* < 0.0001), and stem model (*r* = 0.520; *p* < 0.0001), as it was greater when templating ‘varus’ stems (11.8 ± 2.6 mm) than ‘standard’ stems (9.0 ± 1.9 mm). The cortical distance was weakly correlated with femoral head diameter (*r* = 0.399; *p* < 0.0001), stem size (*r* = 0.200; *p* = 0.017), and patient gender (*r* = 0.361; *p* < 0.0001). It is worth noting that significant associations (*p* < 0.0001) were also found between stem size with both mediolateral offset and femoral head diameter. Furthermore, the mediolateral offset was significantly (*p* < 0.0001) higher in hips templated with varus stems (47.7 ± 4.5 mm) than with standard stems (41.5 ± 4.2 mm).

Multivariable analysis confirmed that stem model (*p* < 0.0001) and femoral head diameter (*p* = 0.0162) are directly correlated to cortical distance. The FNA was neither directly (*p* = 0.5962) nor indirectly (*r* = 0.000; *p* = 0.9288) correlated to cortical distance (Table 2).

**Table 2:** Linear regression analysis of variables associated with Cortical Distance

Variable	Univariable				Multivariable		
	regression coefficient	95% C.I.		*p*-value	regression coefficient	95% C.I.	*p*-value
Continuous							
Age (years)	−0.04	(−1.27	– -0.22)	0.006	−0.02	(−0.04–0.00)	0.093
Medio-lateral offset	0.28	(1.76	– 2.63)	<0.001	0.04	(−0.04–0.11)	0.352
Femoral head diameter	0.30	(1.03	– 1.98)	<0.001	0.14	(0.03–0.26)	0.016
Femoral Neck Angle	0.00	(−0.46	– 0.51)	0.929	0.01	(−0.03–0.06)	0.596
Catagoric							
Stem size							
Small (1–3)	REF				REF		
Medium (4–6)	0.82	(−0.08	– 1.73)	0.075	0.39	(−0.374–1.15)	0.317
Large (7–10)	1.48	(0.47	– 2.50)	0.004	0.93	(−0.031–1.88)	0.058
Varus stem model	2.74	(2.12	– 3.37)	<0.001	2.42	(1.71–3.14)	<0.001
Male Gender	2.11	(−2.87	– -1.35)	<0.001	0.68	(−1.574–0.21)	0.132

## Discussion

The principal findings of the present study were that the cortical distance along the resected femoral calcar is directly correlated with the model of the stem implanted (‘standard’ or ‘varus’) and with the diameter of the femoral head. This cortical distance indicates optimal collar size, which would grant maximum coverage of the femoral calcar, without prosthetic overhang. Our data therefore suggest that collar size should be proportional to the size of the operated hip, and that it should be larger for ‘varus’ stem models than for ‘standard’ stem models. Assimilating dimensions from commercial brochures of different implant manufacturers reveals that only few of them adjust the collar size to the model and size of their femoral stems (Table 3).

**Table 3:** Design characteristics of uncemented collared femoral stems by different manufacturers

Manufacturer	Stem Brand	Femoral neck angle (°)		Stem Size	Collar Size (mm)	
		‘Standard’	‘Varus’		‘Standard’	‘Varus’
Depuy	Corail	135	125	8–10	6	7.5
				11	7	8
				12–14	7	9.5
				15	7	11
				16–20	8	12
Smith and Nephew	Echelon	131	–	11–19	7.5	–
	Synergy	131	–	9–17	7.5	–
Tornier	Meije	130	123	1–3	7	7
				4–6	8	8
				7–10	9	9
Serf	Hype	130	–	1–11^a^	5–7.5	–
Dedienne	Symbol	130	120	1–2	6.5	8
				3–4	7.5	9
				5–6	8.5	10
				7–8	9.5	11
				9–10	10.5	12
Xnov	Cineos	135	125	9–20	7	7

^a^collar size increases by increments of 0.25 mm for each stem size

Numerous studies investigated the benefits and drawbacks of collared stems and found little differences compared to collarless stems (Al-Najjim et al., 2016; Caglar et al., 2008; Ebramzadeh et al., 2004; Lenart et al., 2012; Weber et al., 2014). Other clinical studies encourage the use of collars because it may improve stem survival and simplify revision THA (Flecher et al., 2012; Kale et al., 2000; Van Kleunen et al., 2006). Finite element analyses suggested that collars improve the distribution of axial loads on the femoral calcar and reduce tensile and rotational stresses within the cancellous bone, and thereby reduce risks of fracture and thigh pain (Fischer et al., 1992; Jeon et al., 2011; Whiteside et al., 1988). In case of insufficient support of an uncemented stem within the metaphysis, good collar-calcar coverage could prevent implant subsidence and rotation, and therefore secure its ideal position and osteo-integration. Collarless stems typically subside by 0.5 to 1.5 mm within the first few weeks following THA (Campbell et al., 2011; Parvizi et al., 2004; Simpson et al., 2010; Strom et al., 2007; Weber et al., 2014), which could be limited using collared stems that can withstand twice as much load (Demey et al., 2011; Whiteside et al., 1988). It remains unclear, however, whether collars tend to decrease or increase calcar resorption, as the bone remodelling process depends on multiple factors related to load transfer along the stem surface (Carlsson et al., 1995; Gibbons et al., 2001; Kadar et al., 2011; Sharif & Parker, 2002).

Several authors support that collar efficacy depends on its coverage over the femoral calcar (Demey et al., 2011; Fischer et al., 1992; Jeon et al., 2011; Keaveny & Bartel, 1993; Mandell et al., 2004). Kelley et al.(1993) reported that 4.6 years following THA, 47% of patients had good collar-calcar contact, none of which needed revision. On one hand, an undersized collar may be insufficient to prevent stem subsidence or rotation (Fig. 1a and b). Several stems have relatively small collars that do not reach the medial margin of the femoral calcar and thus bear only on the cancellous bone. In a series of 103 hips implanted with collared stems, Meding et al. (1997) reported insufficient coverage of the femoral calcar by the prosthetic collar in 61% of their patients. On the other hand, an oversized collar could, however, lead to painful ilipsoas impingement against the prosthetic overhang (Fig. 1c). In a case report, Brew et al. (2011) confirmed that, because of a large protruding collar, their patient had iliopsoas tendonitis and required revision surgery. In another case report, Linder et al. (2013) found that iliopsoas tenotomy relieved similar symptoms.

The findings of the present study revealed two interesting trends. The first trend is that cortical distance was correlated to stem size in univariable regression but not in multivariable regression. This is likely because our multivariable model included femoral head diameter, which is more intrinsically correlated with cortical distance, than is the choice of stem size per se. As noted earlier in the results, stem size is most correlated with femoral head diameter, and is thus *indirectly* correlated to cortical distance. Yet when hesitating between two consecutive stem sizes for the same hip, implanting the smaller stem would fill less volume in the femoral metaphysis and leave a greater cortical distance to be covered by the collar. Conversely, implanting the larger stem would fill more volume and therefore leave a smaller cortical distance to be covered by the collar. The second trend is that, while the choice of ‘standard’ or ‘varus’ stem is significantly correlated to cortical distance, in both univariable and multivariable analyses, the FNA is neither directly nor indirectly correlated to collar size. This paradoxical finding could be because the choice of ‘standard’ or ‘varus’ stems is not necessarily dependent on the native FNA, but rather on the restoration of medio-lateral offset, limb length and potential acetabular anomalies. It is worth noting that, in the present study, the authors templated the hips with the sole goal of restoring the centre of rotation of the hip, without much consideration to the acetabulum.

The main strengths of this study are its relatively large sample size (204 hips) and the accurate acquisition of CT-scans in a true frontal view. This study has several limitations related to the population studied and measurement protocol. First, hip templating was performed using one THA stem model, and it is not clear whether our conclusions apply for other commercially available femoral stems. Second, we analysed the dimensions in healthy hips, which do not represent the morphologic characteristics and sizing challenges in arthritic hips. This choice was intended to enable accurate and repeatable calculation of the centre and the diameter of the femoral head without artefacts due to arthritic or congenital deformities. Third, the population studied is predominantly Caucasian white, and may not be representative of other ethnicities. Fourth, the choices of stem size, position and model were made without considering the native acetabulum, which could influence the results. The authors assumed that the head centre corresponds to the articular centre of the hip, which in the authors’ experience is a valid approximation for healthy hips (Schofer et al., 2010). Finally, the method used to measure the cortical distance had an accuracy of 0.5 mm, which may be insufficient considering the small dimensions concerned.

## Conclusions

The present study revealed that femoral cortical distance is correlated to stem model and size. Our findings could help implant manufacturers improve the designs of their existing collared stems to optimise load transfer and prevent iliopsoas impingement. Even if the benefits of collars remain unclear, optimising coverage of the femoral calcar requires adapting collar dimension to patient size and morphology.

## Abbreviations

C-D: Cortical Distance
CT: Computed Tomography
FNA: femoral neck angle
THA: Total Hip Arthroplasty
